# Deciphering Metabolic Pathways in High-Seeding-Density Fed-Batch Processes for Monoclonal Antibody Production: A Computational Modeling Perspective

**DOI:** 10.3390/bioengineering11040331

**Published:** 2024-03-28

**Authors:** Carolin Bokelmann, Alireza Ehsani, Jochen Schaub, Fabian Stiefel

**Affiliations:** 1 Institute of Biochemical Engineering, University of Stuttgart, 70569 Stuttgart, Germany; 2 Boehringer Ingelheim Pharma GmbH & Co.KG, Launch & Innovation, 88400 Biberach an der Riß, Germany; 3 Boehringer Ingelheim Pharma GmbH & Co.KG, Development Biologicals Germany, 88400 Biberach an der Riß, Germany; 4 Boehringer Ingelheim Pharma GmbH & Co.KG, Development Sciences Germany, 88400 Biberach an der Riß, Germany

**Keywords:** intensified fed-batch, mathematical modeling, cellular metabolism, bioprocess optimization, Chinese hamster ovary

## Abstract

Due to their high specificity, monoclonal antibodies (mAbs) have garnered significant attention in recent decades, with advancements in production processes, such as high-seeding-density (HSD) strategies, contributing to improved titers. This study provides a thorough investigation of high seeding processes for mAb production in Chinese hamster ovary (CHO) cells, focused on identifying significant metabolites and their interactions. We observed high glycolytic fluxes, the depletion of asparagine, and a shift from lactate production to consumption. Using a metabolic network and flux analysis, we compared the standard fed-batch (STD FB) with HSD cultivations, exploring supplementary lactate and cysteine, and a bolus medium enriched with amino acids. We reconstructed a metabolic network and kinetic models based on the observations and explored the effects of different feeding strategies on CHO cell metabolism. Our findings revealed that the addition of a bolus medium (BM) containing asparagine improved final titers. However, increasing the asparagine concentration in the feed further prevented the lactate shift, indicating a need to find a balance between increased asparagine to counteract limitations and lower asparagine to preserve the shift in lactate metabolism.

## 1. Introduction

The need for effective (bio-)pharmaceuticals is rising and fast research and development processes become increasingly important to bring innovations rapidly to clinical applications. On the pharmaceutical market, with a value of USD 330.7 in 2021 and increasing to a predicted value of USD 478.08 billion in 2026 [1], monoclonal antibodies (mAbs) are one of the best-selling drugs [2].

To meet the growing needs from industry efficiently, the relevant production processes involving cell-based systems require thoughtful reconsideration and continuous improvements. Chinese Hamster Ovary (CHO) cells are a well-established, predominantly used host system to produce therapeutic mAbs. They are commonly cultivated in fed-batch processes due to easy handling and flexibility [3], with an extensive increase in titer in the last years up to 13 g/L [4,5,6]. Promising approaches for further improvement in the overall productivity of industrial processes, while complying with the high demands towards quality, safety, and efficacy of the product [7], are process intensification using high-seeding-density (HSD) processes and model-based process optimization. Thereby, higher product titers, reduced costs, and improved facility utilization [8] can be achieved. The optimization process relies on various methodologies, including a large number of experiments [7], statistical methods [9,10], or mechanistic approaches that consider the dynamics of the system.

Within the realm of mechanistic understanding, different types of metabolic models exist. Stoichiometric or constraint-based modeling approaches are available that rely on pseudo-steady-state assumptions of intracellular metabolites [10]. For some constraint-based modeling approaches, no knowledge about the dynamics of the system is necessary, which makes them widely applicable and appropriate to be combined with genome-scale models [11], but also limits their usage [7]. To capture the dynamic responses in cell culture processes, information on the system kinetics and mechanistic knowledge can be incorporated into the model. This enables the transition to kinetic metabolic modeling. which is suitable for model-based process optimization, requiring necessary simplifications for computational efficiency and identifiability of parameters.

Recent studies have showcased modeling approaches of different complexity and levels of detail for animal cells like AGE1 and CHO cells (e.g., [7,10,12]) dealing with the mentioned restrictions and requirements. Constraint-based modeling of CHO cells was, for example, used for the characterization of the cellular physiology, the development of media and feeding strategies, the optimization of bioprocess control, and cell engineering [13]. On the other hand, Nolan and Lee conducted pioneer work in kinetic modeling with their work on dynamic modeling of CHO cell metabolism in a standard fed-batch (STD FB) process in 2011. Kinetic rate expressions were defined for the cytosolic reactions, and all other rates were determined by means of metabolic flux analysis [10]. Robitaille et al. built a model describing four different culture conditions including batch and STD FB processes with a single set of parameters using multiplicative Michaelis–Menten equations as kinetic expressions [12]. The more recently published model by Ramos et al. consisted of one segregated cell growth model part and one structured model part of the intracellular metabolism of AGE1.HN.AAT cells producing α1-antitrypsin cultivated in a batch process [7]. One recently developed model by Xing et al. includes the central metabolism of CHO cells as well as the production of the CVA6 VLP vaccine. This model described in detail the growth, death, and lysis of cells, while also including glucose, lactate, glutamine, and ammonia, relying on yields for the description of metabolite changes. The authors were thereby able to predict different conditions in batch cultivations [14]. A hybrid approach was proposed by Monteiro and Kontoravdi [15]. In their study, the authors combined a metabolic network and a reactor model to reduce the need for parameter estimation and increase the predictive power of the model [15]. Another kind of hybrid model was introduced by Okamura et al. [16]. They included three different modules, namely, a kinetic metabolic model until cell death is reached, a data-driven module updating parameters based on changing conditions, and a second kinetic model encompassing protein and DNA synthesis. To our knowledge, only a few groups developed a model of an HSD process, while the other published studies focused on batch and/or STD FB processes. Stepper and co-authors used a mechanistic model to investigate perfusion process data. In their work, the authors described a process intensification that combines a pre-stage perfusion and a high-seeding-density fed-batch production stage. The product titer was increased by 1.9-fold, while keeping the product quality at a comparable level. This could be achieved by optimizing the process, making use of the mentioned mechanistic model and next-generation sequencing [8]. In another study, a flux balance analysis was performed on a genome-based model of CHO cells in an HSD process focusing on the reduced viability in these processes and the effect of a combined addition of lactate and cysteine on the metabolism [17].

The HSD processes differ significantly from those in STD FB processes. The seeding and the overall cell density is higher in the HSD process, while the cell viability decreases more rapidly. Due to the pre-stage perfusion, the cells may enter the production stage in a different cell cycle. Generally, different gene expression patterns were found [8]. Therefore, it is likely that the mechanisms inside the cell might be different between STD FB and HSD processes and might require different model structures and/or parameter sets.

Another challenge in modeling this process is that although CHO cells are well studied as a production system, some important mechanisms are still unknown. The switch in lactate metabolism from its production as part of an inefficient overflow metabolism to its consumption in a more efficient metabolic phase, for example, is a well-known and desired phenomenon that is not yet fully understood mechanistically [18]. Several hypotheses on the cause of this phenomenon exist (see e.g., [18,19,20]) and were included in some models with different strategies based on the introduction of a redox variable [10] or switching function [21], the usage of two different kinetics for the lactate dehydrogenase [7], or simply making the reaction reversible [22].

In this study, we first reconstructed a metabolic network and conducted the constraint-based modeling to provide a better understanding of the system behavior and flux distribution in different culture conditions. Next, we reduced the network and set up kinetic expressions for each reaction, analyzed the model structure, and finally set up an ensemble of dynamic model candidates representing the mechanisms inside CHO cells in HSD processes, including the lactate shift. The initial model fit was evaluated, and further possible improvements and indications on the mechanisms were discussed. Simulation studies were performed to gain further knowledge and evaluate the limitations of the model structure. Optimization strategies for the process were discussed, with a focus on medium optimization and amino acid content.

## 2. Materials and Methods

### 2.1. Cell Line, Cultivation, and Analytics

A suspension CHO cell line was cultivated in intensified fed-batch processes with seeding densities of 10 Million cells/mL (HSD) in 3 L reactors with chemically defined media. To establish these seeding densities, the seed train cultures underwent shake flask processing until the (N-2) stage, followed by an (N-1) pre-stage perfusion as described by Stepper et al. [8]. In the STD FB processes, the same media were used but were seeded with 0.7 million cells/mL. The cells were cultivated for 12 to 14 days in two duplicate runs with 13 sampling points, starting at day zero (D0) at intervals of around 24 h. The feeding occurred continuously via a peristaltic pump. We maintained the glucose concentration above a defined threshold with a bolus feed and introduced antifoam agents whenever necessary.

Alongside the HSD control run, additional runs with lactate and cysteine (LAC + CYS) and bolus medium (BM) addition were performed (based on the work by Krumm et al. [23]). The BM addition took place from day one to six, while lactate addition occurred over the whole cultivation duration, and cysteine was added until day four. For the LAC + CYS supplementation, sodium lactate was added from day 3 to day 13 as a bolus if the concentration fell below 2 to 3 g/L, while cysteine was added as a fixed bolus of 7 mL from day 1 to day 5 [23].

The Cedex HiRes analyzer (Roche Diagnostics, Germany) was employed to ascertain the viable cell density and viability. The quantification of glucose, lactate, ammonia, and the protein concentration (immunoglobulin-G) was conducted using the KonelabTM Prime60i device (Thermo Fisher Scientific Inc., MA, USA). The antibody concentration was determined with a Protein-A HPLC (Thermo Fisher Scientific Inc., MA, USA). For amino acids analytics, except for cysteine, the 7890B GC system (Agilent Technologies, CA, USA) was used. The sample preparation was performed as described by Mohabbat and Drew [24]. Therefore, first, norvaline as an internal standard and an amino acid standard were added to the samples. Purification was then performed via an ion exchange resin solid phase extraction. After elution, the derivatization reagent was added, followed by an organic solvent. To stop derivatization, an acidic reagent was included. The samples were then analyzed via a GC-FID using a Faast amino acid analysis kit (Phenomenex Inc., CA, USA) according to Mohabbat and Drew [24]. For an offset correction, offline pH, pO_2_, and pCO_2_ were measured with a blood gas analyzer (Siemens Health Care, Germany), and pH was monitored online with a glass pH electrode (Hamilton Messtechnik, Germany).

### 2.2. Model Reconstruction and Analysis

For the reconstruction of a central carbon metabolism model, we used the genome-scale model iCHOv1_K1_final developed by Hefzi et al. [11] and the model established by Schaub et al. [25] as references. Supplementary to these, we integrated insights from other central carbon metabolic models [26,27] and information from the databases KEGG [28] for the organism *Cricetulus griseus* (Chinese Hamster), ExPASy [29], and PubChem [30]. All reactions included in the network are given in Table S1 in the Supporting Material.

Conducting the flux balance analysis (FBA) entailed pre-processing steps to convert the concentration measurements to fluxes, identify and remove outliers, and filter the data with the LOWESS (linear locally weighted scatterplot smoother) filtering [31]. The constraints of the flux values were chosen as ±10% of the measured data, and reversibility was included by setting the lower boundaries to either 0 or −1000 for unmeasured fluxes. The objective of the FBA was to maximize the biomass or the product, respectively. Taking the results of the FBA as a starting values of a metabolic flux analysis (MFA), the model was fitted to experimental fluxes to obtain the intracellular flux distribution. Finally, the network was validated (based on R2 > 0.98, results not shown here). For the standard fed-batch (STD FB) process, the FBA included the days 4 to 13 to prevent disturbance due to the lag phase. Given that the HSD process lasted only 11 days, the FBA was, correspondingly, only executed in this period, while for the other processes, it was based on the results of days 2 to 13.

In establishing the kinetic model, our focus was on identifying significant metabolites. We therefore reduced the list of metabolites to the main carbon source glucose, pyruvate as an important intermediate, lactate and ammonia as the pivotal by-products, biomass, and the product (mAb), as well as key amino acids, namely, asparagine, aspartate, glutamate, serine, isoleucine, and leucine. In order to streamline and establish connections between these metabolites, we lumped and simplified the reactions of the metabolic network. Multiplicative Michaelis–Menten equations provided the foundation for the kinetic rates in the model. Kinetic parameters were obtained from literature [12,32,33,34,35,36,37,38], derived from experiments, or chosen arbitrarily. To account for the metabolic shift from lactate production to lactate consumption, diverse hypotheses on the regulation based on literature and experimental results were examined to identify the best-fitting models.

**Figure 1:**
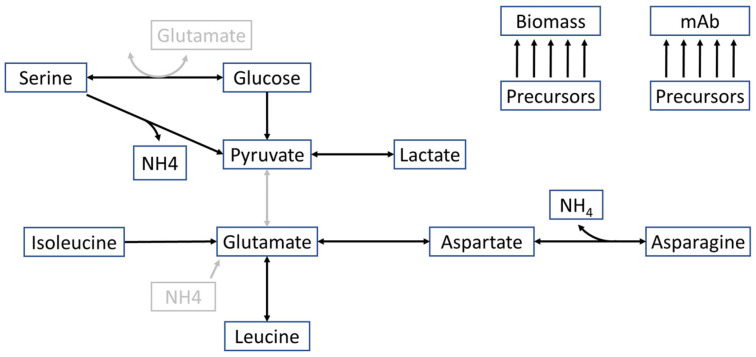
Visualization of the mechanistic model of central carbon metabolism of CHO cells. This figure shows the reactions and metabolites included in the mechanistic metabolic model of CHO cells in the HSD process derived from the metabolic network. Reactions that differ in the different model structures are shown in grey.

We executed the simulations of the kinetic model in MATLAB R2019a (The MathWorks Inc., Natick, MA, USA) by formulating it as a system of ordinary differential equations. For model calibration to the experimental data, we used the enhanced scatter search, readily implemented in the MEIGO toolbox [39]. In pursuit of greater validity, we considered the fluxes observed before and results of a global sensitivity analysis to revise the model structures. Upon observing several well-fitting models, we selected the most suitable models for the given data with the Akaike information criterion [40] and combined those in an ensemble of models. To ascertain the viable parameter space of each model structure, we employed an algorithm combining a coarse-grained global search, the out-of-equilibrium adaptive Metropolis Monte Carlo method, and a finer local search, the multiple ellipsoid-based sampling implemented in the HYPERSPACE toolbox [41]. In the ensemble model, only the five parameter sets yielding the lowest cost function values were incorporated to finally obtain combined predictions. A visualization of the kinetic model structure can be found in Figure 1. The model reactions, kinetics, and parameter sets are listed in the Supporting Material (Tables S3–S5).

## 3. Results

### 3.1. Flux Balance Analysis—Effect of Supplements on the Metabolism

#### 3.1.1. Extracellular Fluxes

**Figure 2:**
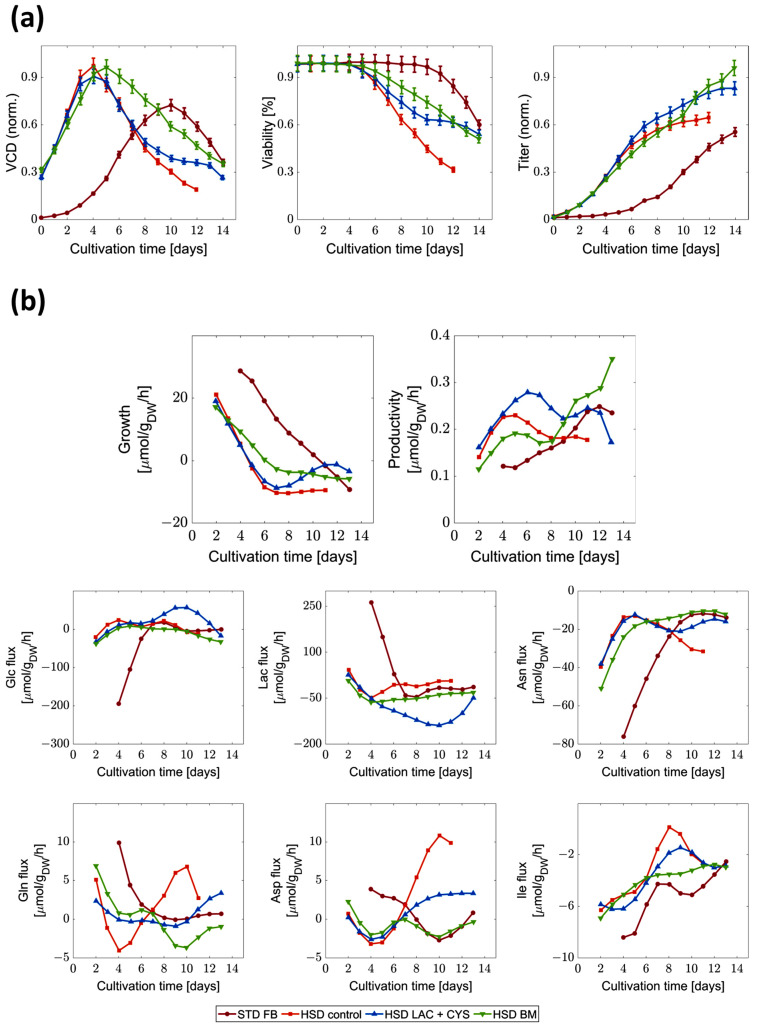
Comparison of viable cell density, viability, titer, and extracellular fluxes between different cultivation conditions. (**a**) Normalized viable cell density (VCD), viability, and titer, as well as (**b**) fluxes derived from concentration measurements for the biomass (growth), the mAb (productivity), glucose (Glc), lactate (Lac), asparagine (Asn), glutamine (Gln), aspartate (Asp), and isoleucine (Ile) formation of the standard fed-batch (STD FB) and high-seeding-density (HSD) processes without additions (control) and with additional lactate and cysteine feed (LAC + CYS) or bolus medium addition (BM) over 12 to 14 days are shown. Negative fluxes indicate consumption while positive fluxes signify secretion.

To better understand the effects of different fermentation conditions, extracellular as well as intracellular fluxes were analyzed. Extracellular fluxes were directly derived from concentration measurements and then used to derive constraints for a flux balance analysis (FBA). Experimental data in Figure 2, as well as previous studies [8,17], revealed that the HSD process increased the overall titer compared to the STD FB process. This improvement was attributed to the HSD process reaching the production phase faster and exhibiting elevated cell-specific productivities [8,17]. However, the viability of the cells dropped after a few days, leading to a decrease in biomass early in the process [23]. To address this issue, the effects of introducing additional lactate and cysteine (LAC + CYS) and bolus medium (BM) were investigated [23] (see Figure 2).

Both LAC + CYS supplementation, as well as the introduction of BM, resulted in 30% increase in viability compared to the control HSD, leading to a higher viable cell density over an extended period. This effect was more pronounced in the run with BM, extending the production phase and resulting in even higher final titers.

Based on these experiments, the FBA was performed (see Figure 2b) to investigate the effect of the additions on the cell metabolism, using the HSD control and LAC + CYS run for calibration and the HSD + BM run for validation.

Comparing the fluxes in STD FB with the HSD process, the STD FB process exhibited higher exponential growth with lower productivity, a characteristic that was shifted to the (N-1) stage in the HSD process. Within the exponential growth phase in the STD FB process, glucose consumption reached higher levels than in the production phase. In the context of this early growth phase, the cells consumed asparagine and isoleucine, while glutamate and aspartate were produced. Remarkably, asparagine consumption was higher than in the other fermentation conditions. Overall, the HSD process showed a different dynamic with divergent trends compared to the STD FB with lower biomass production, higher productivity until day nine, less glucose consumption, and higher lactate production.

Introducing additional BM during day one to six resulted in lower productivity compared to the control condition. However, following the cessation of this addition, productivity notably increased leading to an increased final titer. The addition also slightly increased the lactate uptake while concurrently lowering the glucose uptake and resulted in the uptake of glutamate and aspartate in the second half of the cultivation. Compared to the control HSD condition, the LAC + CYS cultivation strategy exhibited an overall higher productivity with a strong decrease after day 12 (D12). In the initial metabolic phase, the LAC + CYS approach outperformed even the BM addition. Upon addition of lactate and cysteine, lactate uptake increased while the glucose uptake reduced in the subsequent metabolic phase. This observation agrees well with findings in previous studies [42]. The uptake fluxes of amino acids were generally lower than those observed in the BM runs.

During the initial phase of cultivation, a high consumption of asparagine ([µmol/g_DW_/h]) was observed across all conditions, with particularly pronounced levels in the STD FB run. In the STD FB process, large amounts of asparagine were consumed during the lag and growth phase. In the HSD conditions, the main part of the growth phase—and thereby the associated asparagine consumption—was shifted to the (N-1) stage of the cultivation. However, the cells grew exponentially in the first part of the HSD cultivation, leading to high demands for replication and maintenance. This resulted in amino acid limitations and thereby limited uptake rates in the second half of the cultivation. Since additional asparagine was supplied within the BM, the uptake rates sustained higher rates for an extended period leading to the highest maximal viable cell density (see Figure 2). In the LAC + CYS process, where amino acids consumption was generally less extensive, the asparagine consumption remained comparable to that observed in the other processes.

#### 3.1.2. Intracellular Fluxes

The flux distributions derived from FBA, obtained by maximizing the biomass during the cellular growth phase and maximizing mAb production during the production phase, were used for fitting to measurements of metabolites in a metabolic flux analysis. This approach enabled successful validation of the stoichiometric model. Next, we investigated intracellular fluxes by making use of the validated model (see Figure S1 in the Supporting Material).

In the STD FB, the exponential phase was discernible at D4 to D6 by high glycolytic fluxes. These values aligned with the uptake fluxes shown in Figure 2, indicating that a significant portion of glucose was channeled into glycolysis and did not enter the pentose phosphate pathway. After D6, the observed fluxes decreased, which indicated the shift to the production phase.

**Figure 3:**
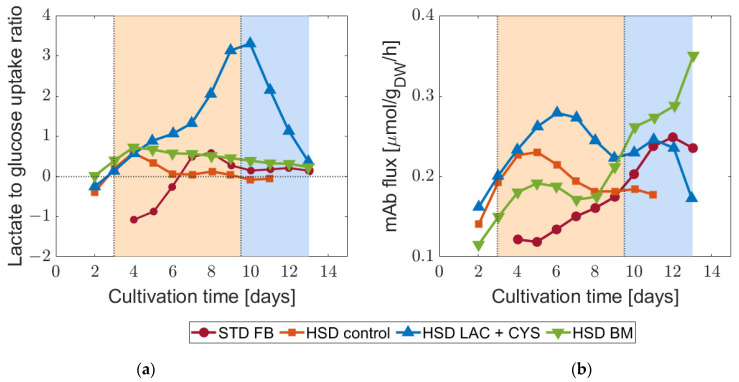
Carbon source and effect on productivity: (**a**) ratio of lactate to glucose determined as the ratio of the uptake fluxes, and (**b**) the mAb productivity accordingly for the standard fed-batch (STD FB) and the three high-seeding-density (HSD) processes. A positive ratio suggests that the cells take up glucose and lactate in parallel, while a negative ratio suggests that glucose is consumed while lactate is produced. The background colors indicate two different metabolic phases.

In the next step, the distinct HSD conditions were compared. The addition of LAC + CYS led to lower glycolytic fluxes from D8 to D11, during which a low glucose level prevailed. Cysteine was only added until D4, so the changes, for example, in the reaction from 3-phosphoglycerate to phosphoenolpyruvate can be attributed solely to lactate. The glycolytic fluxes were generally lower for this condition. For the process with BM addition, the glycolytic activity remained subdued from D4 to D7 and increased afterwards. Directly after D7, the productivity elevated, potentially due to the higher energy provision by glucose uptake. Furthermore, we observed that lactate consumption coincided with low glycolytic activity. To further investigate the role of the carbon source, we calculated the glucose/lactate ratio (see Figure 3)—an indicator of the energetic and metabolic efficiency. A ratio within the range of zero and one indicates a high efficiency [32].

From Figure 3, it is evident that the STD FB process led to an overflow metabolism, where around half of the consumed glucose was flowing into lactate at D4 and D5. Starting from D6 onwards, across the STD FB, HSD control, and HSD + BM conditions, the ratios were between zero and one on most days, indicating glucose as the main carbon source. The addition of the LAC + CYS supplement, on the other hand, led to a ratio exceeding one, signifying lactate as the primary carbon source. This condition led to high mAb productivity. The findings agree with earlier studies [43] wherein lactate was identified as the main carbon source in later stages of cultivation.

All previous results led to the conclusion that the lactate metabolism plays an important role in the production of mAb and its regulation. Therefore, we investigated the individual pathways involved in this part of the metabolism in more detail. Trying to identify a certain switching point in metabolism during the cultivation, the observations from intracellular and extracellular fluxes led to a shift at D7 or D9, respectively. These demarcations were determined based on the ratio between TCA cycle and glycolysis fluxes. In the initial phase, anaplerosis promoted lactate formation, whereas in the subsequent phase, the process shifted to lactate consumption with a more reliable carbon transfer into the TCA cycle. Further details regarding this phenomenon are discussed in Section 4.

These results suggest a connection between the lactate shift and the interplay between the glycolysis and the TCA cycle. Next, we used the findings presented to set up a kinetic model to study a further feeding strategy and present one plausible implementation of the influences revealed so far.

### 3.2. Ensemble Model Calibration

For setting up a kinetic mechanistic model, regulations drawn upon diverse sources from the literature on different organisms and from experimental data were tested. The most probable models according to the data also considering the number of parameters were selected. The three most probable models identified all relied on the same regulation of the lactate shift. This framework encompasses the activation of glycolysis by asparagine, feedback inhibition of lactate dehydrogenase by the product lactate, a link between decreasing glycolysis and increasing lactate uptake, and an inhibition of the entry of pyruvate into the TCA by asparagine (see Figure S2 in the Supporting Material). It is notable that the inclusion of extra asparagine alongside the BM led to an increase in lactate uptake, which gave a first indication that asparagine might be involved in the regulation of the shift. Therefore, we included this kind of regulation. Further discussion around it follows in Section 4.

**Figure 4:**
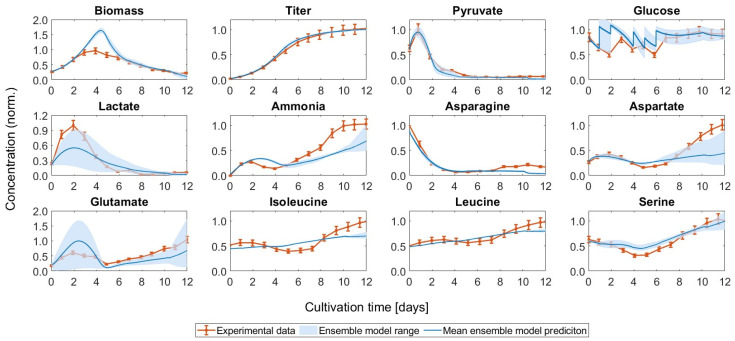
Prediction of the ensemble of models for the control run. The combined predictions are given as the means of the individual model predictions. The whole range of predictions of the models in the ensemble is also visualized for all metabolites and states included in the models.

As described in the method section, an ensemble of models was set up. Combining the selected model structures and parameter sets for the kinetic models, the combined predictions shown in Figure 4 were found. For most metabolites, all models predicted a similar development. Only for lactate, glutamate, and aspartate, the discrepancies were larger.

All models overestimated the biomass concentration at the peak. The lactate shift, although underestimated in some cases, could be reproduced trend-wise by all of the models. The same observation applies to glutamate, where discrepancies were also apparent in the final concentrations. For the combined prediction, which corresponds to the average of the individual model predictions, Pearson’s linear correlation coefficients were between 0.553 for glutamate and 0.998 for mAb, indicating a satisfactory fit.

### 3.3. Room for Improvement—The Lactate Shift

As one important indicator of metabolic efficiency, influencing the productivity, inhibition, and connectivity of metabolic pathways, lactate is of central importance for the metabolism of CHO cells. The mechanistic understanding of the shift from lactate production in the context of inefficient overflow metabolism to the more efficient consumption and utilization as a carbon source remains incomplete. Insights gleaned through the FBA unveiled that lactate consumption is interconnected with the glycolytic activity, the connectivity of the glycolysis and the TCA cycle, and the uptake of several other metabolites including amino acids. In our ensemble model, we modeled the lactate shift by combining these regulations. The derived metabolic expression is given in the Supporting Material Equations (S16), (S17), and (S24) for model 1, as well as in the according rate expressions of the other models. To incorporate the feedback inhibition of the conversion of pyruvate to lactate, an inhibition kinetic with a parametric exponent was chosen. Additionally, the rate from lactate to pyruvate is inversely proportional to the glycolytic rate (S17). This rate is limited not only by glucose but also by asparagine (S16). The further metabolism of pyruvate is also influenced by asparagine, represented by an inhibition (S24).

As described in the existing literature, during the initial growth phase, metabolic inefficiency prevails due to the high uptake of glucose and production of lactate, a waste product that has inhibitory effects. This pathway is further discussed in the subsequent section. By implementing elevated seeding densities for the process, lactate production was reduced markedly in our experiments. Concurrently, lactate was consumed in later cultivation phases.

Several studies have delved into the lactate shift, giving hypotheses on the mechanisms behind it (refer, for instance, to [10,18,19,20,44,45,46,47]). In the process of model development undertaken in this study, a range of hypotheses on the mechanism was implemented. The hypothesis selected as the most probable here has, to our knowledge, never been stated in this combination before. It includes the combined effect of asparagine on glycolysis and on the link between glycolysis and the TCA cycle with the inhibition of the lactate dehydrogenase. We were able to reproduce the lactate shift with these mechanisms. Making use of dynamic metabolic models like the one constructed here facilitates model-based process optimization. Since we assume a large impact of asparagine on the lactate shift, the effect of changed asparagine concentrations in the feeding media was investigated using the model and further measurements.

### 3.4. Effect of Changing Media Composition on the Metabolism of CHO Cells

**Figure 5:**
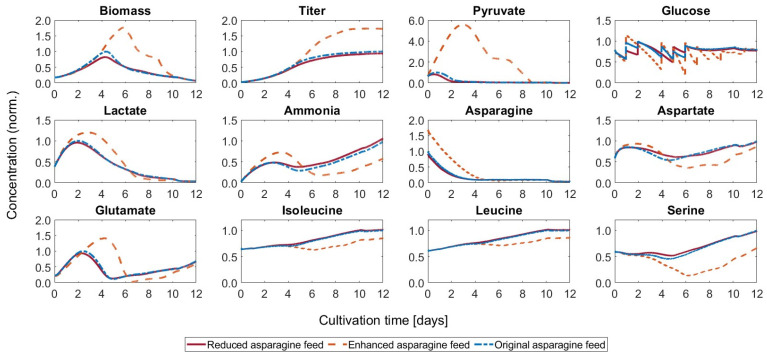
Comparison of the concentration curves for cultivations with different asparagine ratios in the feed media. The simulated development of the concentrations for the original feeding medium, a feeding medium with reduced asparagine concentration, and a feeding medium with elevated asparagine concentration compared to the original medium are shown.

We were able to demonstrate that the addition of a BM containing asparagine improved the performance of the HSD process. Building upon this advancement, the next logical progression involves exploring the potential of a bolus medium with even higher amino acid concentrations. Since asparagine decreased strongly in the beginning and was almost depleted, the investigation in this study focuses on asparagine. For this purpose, we harnessed our model to predict the influence of changing asparagine concentrations in the feed (see Figure 5).

**Figure 6:**
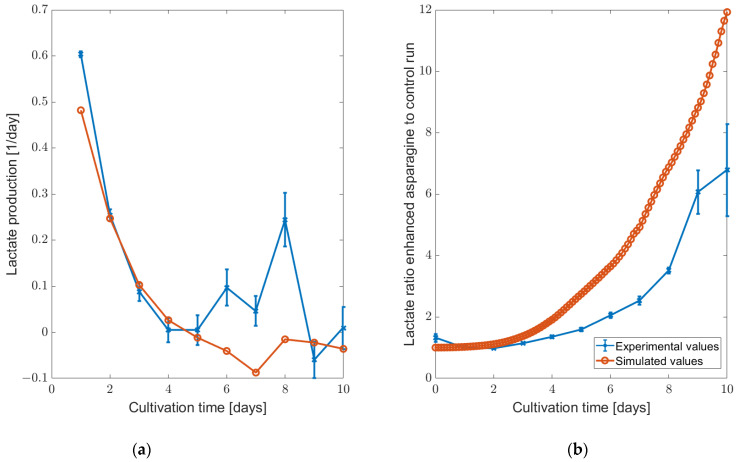
Comparison of enhanced and control asparagine feed in the experiments and simulations based on (**a**) lactate production and (**b**) lactate concentration ratio in the HSD setting.

When reducing the asparagine concentration in the feeding medium, we noted only marginal changes in the concentration profiles predicted by the ensemble model compared to the unchanged feeding medium (refer to Figure 5). While there was a slight alteration in biomass development, it should be noted that the model could not predict this state stably, so this change is difficult to analyze. Additionally, lactate depletion occurred earlier due to the more pronounced shift to lactate consumption. However, the trend of the asparagine concentration curve remained unchanged. Given that only a modest reduction in asparagine concentration was applied, only small changes were to be expected. On the other hand, when increasing the asparagine concentration by a larger percentage, the lactate shift did not occur. The validation experiments that were conducted based on these findings could verify this phenomenon in the cell; with increased asparagine in the feed, no lactate switch was observed (see Figure S4 in the Supporting Material). For further validation of the results, the ratios between the lactate concentration in the enhanced asparagine and control run for the exponential growth phase and production phase (D0 to D10) of the simulations and the experiments were compared, as well as the lactate production rates (see Figure 6). The comparisons showed good agreement with the experiments.

For further analysis, the reaction rates of the kinetic model for the original, higher, and lower asparagine content in the feed were extracted and analyzed on D2 and D5, with time points marking different metabolic phases [23] (see Figure S3 in the Supporting Material). Lower asparagine content in the feed fostered a more pronounced lactate shift reducing the inhibition by lactate, accompanied by an enhanced growth rate. Higher asparagine reduced limitations and depletion of important amino acids, also resulting in elevated growth. The production of mAb was predicted to be higher with enhanced asparagine level. However, this contradicts experimental findings (see Figure S4 in the Supporting Material). Notably, the rates once again showed that lactate production did not switch to lactate production for the simulations with higher asparagine content in the feed.

## 4. Discussion

In this study, the metabolic behavior of CHO cells underwent deeper investigation using experimental and computational tools. One distinct characteristic of CHO cell metabolism during the exponential growth phase is the metabolic overflow, commonly referred to as the Warburg effect [48], resulting in high lactate production. This path only yields 2 ATP molecules, while the complete oxidation in the TCA cycle fueling the oxidative phosphorylation leads to an ATP gain of 36 molecules [49,50]. As mentioned before, under certain conditions, the metabolism can switch to a more efficient state. In that state, glycolysis rates are low, and lactate is produced less [49,51], or even consumed [44,49]. In the context of the mAb production process, the lactate shift is highly desirable if the lactate production itself cannot be avoided in the first place since the reduction of extracellular lactate reduces inhibitory effects, leading to higher viable cell densities and product yields [49]. Additionally, the decrease in the pH by high extracellular lactate concentrations, or the increase in the osmolality in a pH-controlled environment by addition of a base to counteract the pH decrease [44], is reduced. Strategies to enhance CHO cell metabolism involve either preventing this glucose metabolism route or shifting this part from the production stage to the (N-1) stage of fermentation as was undertaken in the HSD condition.

In addition to the control HSD condition, fermentation runs with additional lactate and cysteine and an additional BM were conducted. In a previous study in the same group by Brunner et al., it was shown that the introduction of lactate aimed to increase productivity and reduce reactive oxygen species (ROS) formation. Cysteine, typically added to maintain cell growth, exhibited the capability to actively reduce ROS [17] and might also have an influence on the glycolytic and TCA cycle activity [48] and product quality [42]. The BM contained a combination of amino acids thought to reduce limitations. Higher lactate, glutamate, and aspartate uptake in the HSD + BM cultivation led to higher carbon availability, which had a positive effect on the productivity in that condition. The observed impact of BM could potentially also be attributed to a reduction in DNA damage and apoptosis by the presence of higher amounts of asparagine and higher transport activities triggered by higher isoleucine concentrations that prevents the depletion of other amino acids [32,52]. In the LAC + CYS cultivation, the amplification of lactate uptake, leading to high amounts of pyruvate entering the mitochondrion and the TCA cycle, might explain the lower uptake fluxes of amino acids since these are not necessary to fulfill carbon requirements. This phenomenon aligned with the outcomes of the FBA.

The high asparagine consumption across all conditions in the first phase of cultivation was one important finding. From the results of the FBA, considering the additional BM, one possible conclusion is that asparagine is limiting in the HSD cultivations. Therefore, if more asparagine were available, the growth phase would be extended. Additionally, under these conditions, cells co-consumed asparagine, glutamate, and aspartate in the later cultivation phase to fulfill the lifted carbon demand for enhanced productivity. Overall, asparagine appeared to be a critical metabolite for the cells.

Further analysis of intracellular fluxes revealed a potential connection between glycolytic activity and the lactate shift. In the initial phase, anaplerosis was active, manifesting in the generation of lactate and the entering of pyruvate into the TCA cycle via oxaloacetate and malate. This phenomenon signifies overflow metabolism and an inefficient usage of pyruvate. In the subsequent phase, lactate was taken up and pyruvate could directly enter the mitochondrion, leading to a more reliable carbon transfer in the TCA cycle. It is worth noting that anaplerosis energetically involves ATP expenditure for the conversion of pyruvate into oxaloacetate. One reason for the use of this pathway could be the cytosolic accumulation of lactate before its export inducing an acidic milieu that might have hindered pyruvate transport through the mitochondrial membrane due to a diminished potential across this membrane [53]. Lactate consumption and utilization within the cell could have facilitated the restoration of the membrane potential. Compared to the STD FB approach, the HSD process improved the efficiency and led to less anaplerosis. In the STD FB process, higher glycolytic fluxes are present while having less and later lactate consumption. The most pronounced lactate consumption flux was observed for the LAC + CYS, which, at the same time, showed the lowest glycolytic fluxes. In the same condition, the lactate-to-glucose-uptake ratio showed significant differences compared to the other runs. The higher availability of lactate in the medium led to the shift to lactate as the main carbon source although glucose was still present. This run resulted in higher titers than the control HSD cultivation, so one might conclude that processes solely relying on lactate might be an option for future cultivations. However, CHO cells cannot solely rely on lactate as a carbon source since glycolytic intermediates also contribute to other metabolic pathways, so cells abstain from lactate consumption in the absence of glucose [54].

Considering the results of the cultivation runs with LAC + CYS and BM addition, further optimization of the feeding strategy in the process is possible. Notably, lactate was highly consumed until D9 in the LAC + CYS case and at the same time point, the BM condition surpassed the LAC + CYS condition in terms of mAb productivity. Finding an appropriate way to combine these two processes, the benefits of each condition could lead to an even higher titer.

After intensively studying the fluxes in the metabolism of CHO cells, a kinetic model was constructed. The presented mechanisms for the regulation of the metabolism within the organisms cannot be definitively asserted. The hypothesis, albeit constructed based on diverse literature findings across various organisms, emerged as the most plausible among the range of hypotheses tested. It was notable that the inclusion of extra asparagine alongside the BM led to an increase in lactate uptake, which gave a first indication that asparagine might be involved in the regulation. In the FBA, we could observe high asparagine uptake rates at the beginning in parallel to lactate production. Further insights emerged from concentration measurements, unveiling a concurrent reduction in asparagine levels alongside the occurrence of the lactate shift. While this does not definitively demonstrate causation, it might give a further indication on the regulation mechanism. Findings by Pan et al. support this conclusion [20]. They reported an inhibition of the reaction from lactate to pyruvate by asparagine. The activation of the glycolysis by asparagine can be derived from the activation of the enzyme hexokinase by TCA cycle intermediates in yeast [55,56], which can be provided by asparagine. The feedback inhibition of lactate dehydrogenase by lactate was described before [57], and it was indicated that lactate consumption could be triggered by low glycolytic rates [58]. Furthermore, the hypothesis that the entry of pyruvate into the TCA cycle is inhibited by asparagine was deduced from the inhibition of pyruvate dehydrogenase in pig by TCA cycle intermediates [59], which, once again, can be provided by asparagine. An overview of the different activations and inhibitions is given in Figure S2 in the Supporting Material. The fact that the lactate switch could be accurately predicted using this regulatory framework suggests its applicability within the scope of our purposes.

The constructed model was further used for simulation studies and was the foundation for extended experiments. The most interesting finding was that when increasing the asparagine concentration by a larger percentage, the lactate shift did not occur. This means that a compromise between increased asparagine to prevent limitations and lower asparagine to prevent its effect on the lactate metabolism is needed. Due to the mechanisms implemented in the model, an increase in asparagine led to an elevated glycolytic rate that, in turn, inhibits lactate consumption. At the same time, less pyruvate enters the TCA cycle, leading to an accumulation of pyruvate that shifts the equilibrium of the reaction from pyruvate to lactate to the side of lactate. Experiments could verify this phenomenon, indicating that the regulation assumed here might indeed hold relevance in the biological context. In contrast to the simulation results, in the experimental values, no increased final titer was observed for enhanced asparagine feed, leading to the conclusion that relevant regulations involved in mAb production might be missing in the model. This regulation could be connected to the extracellular lactate concentration that was increased due to elevated asparagine concentrations. The higher availability of asparagine and the higher inhibition by lactate might balance each other out, leading to an unchanged final titer. In contrast to these findings, Lao and Toth reported that increased lactate concentration elevated the specific productivity slightly [60]. However, the increased osmolarity, which comes with higher lactate concentration in a pH-controlled environment, was reported to decrease the protein production [60]. Cruz et al. also found a decrease in productivity attributed to the combined effect of elevated lactate and higher osmolarity [61]. These findings underscore the necessity of incorporating a regulation governing mAb production that accounts for regulation by lactate and/or osmolarity. The further improvement of the ensemble of models is out of the scope of this study, but the findings here can pave the way for more reliable models in the future.

## 5. Conclusions

With a combination of experimental and computational tools, in-depth analysis of metabolism in high-seeding-density fed-batch processes for mAb production was conducted. We developed a stoichiometric and an ensemble of kinetic models of CHO cell central carbon metabolism, validated them, and analyzed them under standard and high-density conditions with various feeding strategies. Our kinetic model, rooted in an interplay of glycolytic activity, lactate feedback inhibition, and asparagine influence, aimed to reproduce the lactate metabolism shift. Our study focused on diverse cultivation modalities and feeding strategies, revealing increased titers under high-density conditions and additional lactate/cysteine or bolus medium feed. These additives improved lactate utilization, enhancing metabolic efficiency. However, contrary to predictions, highly increased asparagine did not elevate product titers endlessly, suggesting additional not modeled regulatory mechanisms, possibly involving lactate or osmolarity.

Upon identifying a model capable of projecting the influence of the media composition on the metabolism, it becomes a valuable tool for further process development. Given that its predictions are solely based on extracellular measurements, a straightforward integration of the model into a digital twin of the process is feasible. This enables real-time predictions of concentration changes, providing insights into the ongoing process and allowing prompt adaptations if necessary.

While the models developed in this study have advanced our understanding of CHO cell metabolism, there remains an opportunity for further refinement and exploration. Future work should include additional iterations of experiments, revision of model structure, and model calibration to enhance the accuracy and predictive power of the models. Moreover, a more granular investigation into the relationship between asparagine and the lactate shift could yield valuable insights, potentially unlocking new paths for process optimization. This iterative and investigative approach will not only deepen our understanding of the complex mechanisms at play but also provide a robust foundation for the continual improvement of bioprocesses. It is important to note that this work did not consider the variations in product quality. Therefore, a significant step in future studies would be to investigate how these variations influence quality, providing another layer of depth to our understanding and optimization of the bioprocess.

## Data Availability

Data are contained within the article.

## Supplementary Materials

The following supporting information can be downloaded at https://www.mdpi.com/article/10.3390/bioengineering11040331/s1. Table S1: List of reactions in the stoichiometric metabolic network; Table S2: List of abbreviations of metabolites in the models; Table S3: List of reactions in the kinetic central carbon models; Table S4: List of ordinary differential equations and rate expressions for the three distinct models; Table S5: List of nominal and optimized kinetic parameters with reference value and boundaries; Figure S1: Heatmap of intracellular metabolic fluxes; Figure S2: Visualization of the regulations behind the lactate shift in the dynamic model; Figure S3: Comparison of rates for different asparagine concentrations in the feeding media on day two and day five of the cultivation; Figure S4: Measurements for experiments with the original and enhanced asparagine feed.
