# Supplementary material for: Deciphering Metabolic Pathways in High-Seeding-Density Fed-Batch Processes for Monoclonal Antibody Production: A Computational Modeling Perspective
Source: Bioengineering (Basel). 2024 Mar 28;11(4):331. doi: 10.3390/bioengineering11040331 (PMC11048072; doi:10.3390/bioengineering11040331)
Supplement: Supplementary file 1 [file bioengineering-11-00331-s001.zip › bioengineering-2908446-Table S5.pdf]

Table S5. List of nominal and optimized kinetic parameters with reference value and boundaries.

Parameter	Nominal	Ref.	Bounds	Optimized model 1	Optimized model 2	Optimized model 3
$v_{max,1}$	$7.000 \cdot 10^{-2}$	exp., [33]	10 % - 5 %	$9.840 \cdot 10^{-2}$	$1.185 \cdot 10^{-1}$	$1.117 \cdot 10^{-1}$
$v_{max,3}$	$7.000 \cdot 10^{-1}$	exp.	10 % - 5 % (only upper bound)	$1.014 \cdot 10^{0}$	$5.082 \cdot 10^{-1}$	$8.679 \cdot 10^{-1}$
$v_{max,4f}$	$7.000 \cdot 10^{-1}$	exp.	50 % - 25 %	$1.067 \cdot 10^{0}$	$8.698 \cdot 10^{-1}$	$7.554 \cdot 10^{-1}$
$v_{max,4r}$	$2.000 \cdot 10^{-1}$	exp.	50 % - 25 %	$1.897 \cdot 10^{-1}$	$2.987 \cdot 10^{-1}$	$8.570 \cdot 10^{-2}$
$v_{max,5f}$	$1.900 \cdot 10^{-1}$	exp.	50 % - 25 %	$2.363 \cdot 10^{-1}$	$2.029 \cdot 10^{-1}$	$2.210 \cdot 10^{-1}$
$v_{max,5r}$	$1.000 \cdot 10^{-3}$	exp.	50 % - 25 %	$1.098 \cdot 10^{-5}$	$3.656 \cdot 10^{-5}$	$2.150 \cdot 10^{-2}$
$v_{max,6f}$	$7.800 \cdot 10^{-1}$	[12]	75 % - 50 %	$2.356 \cdot 10^{-1}$	$1.249 \cdot 10^{0}$	$1.579 \cdot 10^{0}$
$v_{max,6r}$	$1.000 \cdot 10^{-3}$	arb.	75 % - 50 %	$1.962 \cdot 10^{-1}$	$3.306 \cdot 10^{-1}$	$1.695 \cdot 10^{-1}$
$v_{max,7}$	$7.000 \cdot 10^{-1}$	exp.	10 % - 5 %	$9.370 \cdot 10^{-2}$	$2.137 \cdot 10^{-1}$	$9.890 \cdot 10^{-2}$
$v_{max,8}$	$2.000 \cdot 10^{-2}$	arb.	50 % - 25 %	$5.100 \cdot 10^{-3}$	$2.100 \cdot 10^{-3}$	$9.800 \cdot 10^{-3}$
$v_{max,9f}$	$4.800 \cdot 10^{-2}$	arb.	50 % - 25 %	$2.690 \cdot 10^{-2}$	$2.220 \cdot 10^{-2}$	$2.670 \cdot 10^{-2}$
$v_{max,9r}$	$1.000 \cdot 10^{-3}$	arb.	75 % - 50 %	$8.287 \cdot 10^{-5}$	$1.326 \cdot 10^{-4}$	$3.489 \cdot 10^{-4}$
$v_{max,10}$	$8.500 \cdot 10^{-1}$	[12]	75 % - 50 %	$9.909 \cdot 10^{-1}$	$7.040 \cdot 10^{-2}$	$7.420 \cdot 10^{-2}$
$v_{max,11f}$	$3.900 \cdot 10^{-1}$	[12]	75 % - 50 %	$5.372 \cdot 10^{-1}$	$4.674 \cdot 10^{-1}$	$5.078 \cdot 10^{-1}$
$v_{max,11r}$	$1.300 \cdot 10^{-1}$	arb.	75 % - 50 %	$2.147 \cdot 10^{-1}$		
K_{mpyr1}	$3.900 \cdot 10^{-2}$	[12]	75 % - 50 %	$8.290 \cdot 10^{-2}$	$7.300 \cdot 10^{-3}$	$8.000 \cdot 10^{-3}$
K_{mglu1}	$1.700 \cdot 10^{-2}$	[12]	75 % - 50 %	$3.717 \cdot 10^{-4}$	$7.994 \cdot 10^{-1}$	$7.562 \cdot 10^{-1}$
K_{mglc1}	$7.500 \cdot 10^{-1}$	[33]	75 % - 50 %	$1.084 \cdot 10^{-1}$	$9.030 \cdot 10^{-2}$	$1.269 \cdot 10^{-1}$
K_{masn1}	$4.200 \cdot 10^{-3}$	[12]	75 % - 50 %	$2.984 \cdot 10^{-5}$	$1.180 \cdot 10^{-2}$	$1.600 \cdot 10^{-3}$
K_{dnh41}	$1.500 \cdot 10^{+1}$	[33]	75 % - 50 %	$3.450 \cdot 10^{+2}$	$3.449 \cdot 10^{+1}$	$2.843 \cdot 10^{+1}$
K_{dlac1}	$9.000 \cdot 10^{+1}$	[33]	75 % - 50 %	$1.171 \cdot 10^{+3}$		
K_{mglc3}	$1.100 \cdot 10^{-1}$	[32]	75 % - 50 %	$7.400 \cdot 10^{-3}$	$1.700 \cdot 10^{-3}$	$4.600 \cdot 10^{-3}$
K_{masn3}	$4.000 \cdot 10^{0}$		75 % - 50 %	$4.463 \cdot 10^{0}$	$1.507 \cdot 10^{0}$	$2.965 \cdot 10^{0}$
K_{mpyr4}	$3.000 \cdot 10^{-2}$	[32]	75 % - 50 %	$2.065 \cdot 10^{-4}$	$2.800 \cdot 10^{-3}$	$2.910 \cdot 10^{-1}$
K_{mlac4}	$1.790 \cdot 10^{0}$	[32]	75 % - 50 %	$1.809 \cdot 10^{-1}$	$2.457 \cdot 10^{0}$	$5.473 \cdot 10^{-1}$
K_{ilac4}	$2.000 \cdot 10^{+1}$		75 % - 50 %	$3.491 \cdot 10^{+1}$	$6.268 \cdot 10^{0}$	$1.613 \cdot 10^{+1}$
K_{masn5}	$2.240 \cdot 10^{0}$	[34]*	75 % - 50 %	$9.946 \cdot 10^{-1}$	$6.989 \cdot 10^{-1}$	$6.893 \cdot 10^{-1}$
K_{masp5}	$4.000 \cdot 10^{-2}$	[12]	75 % - 50 %	$1.800 \cdot 10^{-2}$	$3.350 \cdot 10^{-2}$	$5.300 \cdot 10^{-2}$
K_{mnh45}	$1.100 \cdot 10^{0}$	[12]	75 % - 50 %	$8.172 \cdot 10^{-1}$	$2.050 \cdot 10^{0}$	$2.632 \cdot 10^{0}$
K_{masp6}	$5.000 \cdot 10^{-1}$	[35]*	75 % - 50 %	$1.336 \cdot 10^{0}$	$2.996 \cdot 10^{0}$	$7.696 \cdot 10^{0}$
K_{mglu6}	$7.500 \cdot 10^{-1}$	[12]	75 % - 50 %	$1.955 \cdot 10^{0}$	$3.700 \cdot 10^{-2}$	$5.000 \cdot 10^{-3}$
K_{mglu7}	$7.500 \cdot 10^{-1}$	[12]	75 % - 50 %	$2.439 \cdot 10^{-1}$		
K_{mglc7}	$2.000 \cdot 10^{+1}$	[12]	75 % - 50 %	$4.737 \cdot 10^{0}$	$7.417 \cdot 10^{+1}$	$3.776 \cdot 10^{+1}$
K_{mile8}	$2.220 \cdot 10^{+1}$	[36]*	75 % - 50 %	$6.862 \cdot 10^{0}$	$5.027 \cdot 10^{0}$	$5.611 \cdot 10^{0}$
K_{mleu9}	$1.110 \cdot 10^{+1}$	[36]*	75 % - 50 %	$1.496 \cdot 10^{0}$	$7.041 \cdot 10^{-1}$	$1.948 \cdot 10^{-1}$
K_{mglu9}	$6.650 \cdot 10^{0}$	[37]*	75 % - 50 %	$1.273 \cdot 10^{0}$	$5.398 \cdot 10^{0}$	$1.578 \cdot 10^{0}$
K_{mser10}	$4.500 \cdot 10^{+1}$	[38]*	75 % - 50 %	$1.203 \cdot 10^{+2}$	$5.354 \cdot 10^{0}$	$1.364 \cdot 10^{+1}$
k_d	$4.500 \cdot 10^{-2}$	[33]	75 % - 50 %	$6.830 \cdot 10^{-2}$	$7.300 \cdot 10^{-2}$	$6.360 \cdot 10^{-2}$
m_{glc}	$1.780 \cdot 10^{-1}$	[33]	75 % - 50 %	$4.250 \cdot 10^{-2}$	$3.380 \cdot 10^{-2}$	$4.130 \cdot 10^{-2}$
K_{mpyr11}	$3.900 \cdot 10^{-2}$	[12]	75 % - 50 %	$2.803 \cdot 10^{-4}$	$2.346 \cdot 10^{-1}$	$2.986 \cdot 10^{-1}$
K_{iasn11}	$4.000 \cdot 10^{0}$		75 % - 50 %	$2.338 \cdot 10^{0}$	$2.569 \cdot 10^{+1}$	$5.383 \cdot 10^{0}$
a_2	$1.000 \cdot 10^{-4}$		75 % - 50 %	$1.772 \cdot 10^{-4}$	$1.847 \cdot 10^{-4}$	$1.834 \cdot 10^{-4}$
b_2	$3.000 \cdot 10^{-3}$		75 % - 50 %	$1.289 \cdot 10^{-6}$	$1.879 \cdot 10^{-5}$	$4.804 \cdot 10^{-5}$
γ_4	$3.000 \cdot 10^{0}$		75 % - 50 %	$4.006 \cdot 10^{0}$	$4.544 \cdot 10^{-1}$	$3.710 \cdot 10^{0}$
$v_{3,base}$	$1.000 \cdot 10^{-1}$		75 % - 50 %	$4.010 \cdot 10^{-2}$	$1.345 \cdot 10^{-1}$	$6.190 \cdot 10^{-2}$
$Y_{lac/biomass}$	$1.670 \cdot 10^{+1}$		75 % - 50 %			$3.233 \cdot 10^{+2}$

* Kinetic parameters found on BRENDA originate from data for the species Rattus norvegicus or Cavia porcellus since no data was available for Cricetulus griseus (Chinese hamster); BRENDA is available at www.brenda-enzymes.org;
exp. = observed maximal fluxes, arb. = arbitrary values
