# Supplementary material for: Deciphering Metabolic Pathways in High-Seeding-Density Fed-Batch Processes for Monoclonal Antibody Production: A Computational Modeling Perspective
Source: Bioengineering (Basel). 2024 Mar 28;11(4):331. doi: 10.3390/bioengineering11040331 (PMC11048072; doi:10.3390/bioengineering11040331)
Supplement: Supplementary file 1 [file bioengineering-11-00331-s001.zip › Supporting Material Figure S1.pdf]

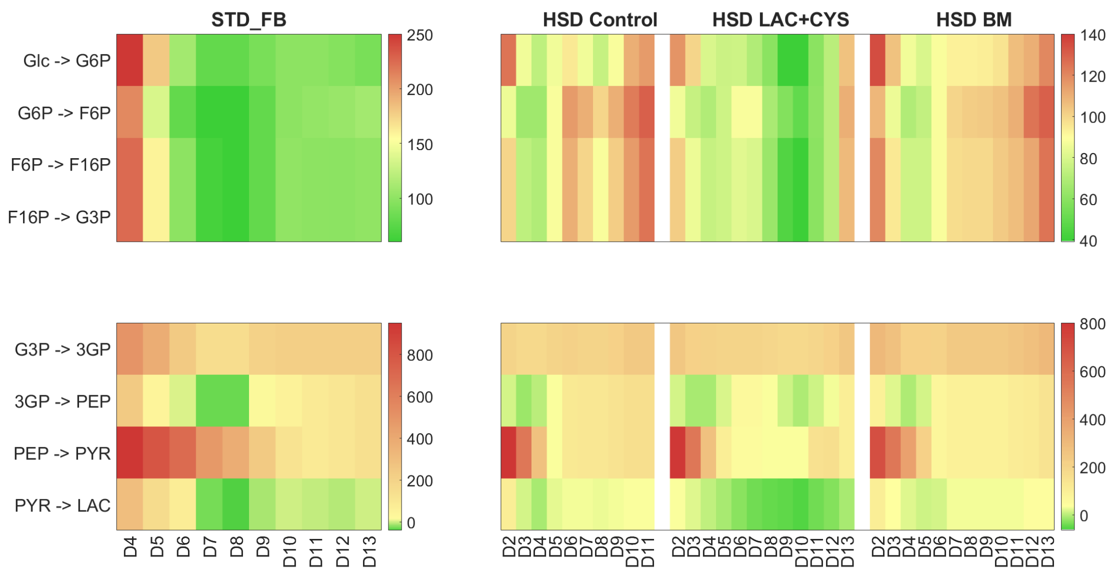

Figure S1. Heatmap of intracellular metabolic fluxes. Glycolytic fluxes [μmol/gDW/h] for the standard fed-batch process (STD FB) from day four to day 13 (not considering the lag phase) and the high seeding density (HSD) processes without further additions (Control), with additional lactate and cysteine feed (LAC/CYS) and with additional bolus medium feed (+BM) from day two to eleven or thirteen are shown, respectively.
