# Supplementary material for: Deciphering Metabolic Pathways in High-Seeding-Density Fed-Batch Processes for Monoclonal Antibody Production: A Computational Modeling Perspective
Source: Bioengineering (Basel). 2024 Mar 28;11(4):331. doi: 10.3390/bioengineering11040331 (PMC11048072; doi:10.3390/bioengineering11040331)
Supplement: Supplementary file 1 [file bioengineering-11-00331-s001.zip › Supporting Material Figure S3.pdf]

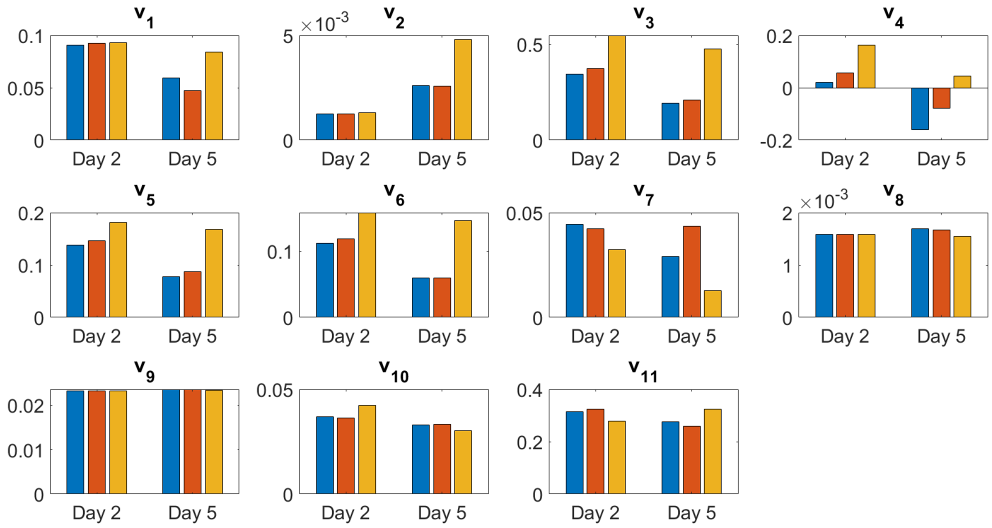

Figure S3. Comparison of rates for different asparagine concentrations in the feeding media on day two and day five of the cultivation. The rates are given for day two and day five for the feeding media with the original asparagine portion (orange), reduced asparagine (blue), and elevated asparagine (yellow) for the eleven reactions (v1: biomass generation, v2: mAb production, v3: glycolysis, v4: lactate production (positive)/consumption (negative), v5: asparagine to aspartate, v6: aspartate to glutamate, v7: glutamate to serine, v8: isoleucine to glutamate, v9: leucine to glutamate, v10: serine to pyruvate, v11: pyruvate to glutamate, see Supporting Material Table S4).
