# Supplementary material for: Deciphering Metabolic Pathways in High-Seeding-Density Fed-Batch Processes for Monoclonal Antibody Production: A Computational Modeling Perspective
Source: Bioengineering (Basel). 2024 Mar 28;11(4):331. doi: 10.3390/bioengineering11040331 (PMC11048072; doi:10.3390/bioengineering11040331)
Supplement: Supplementary file 1 [file bioengineering-11-00331-s001.zip › Supporting Material Figure S4.pdf]

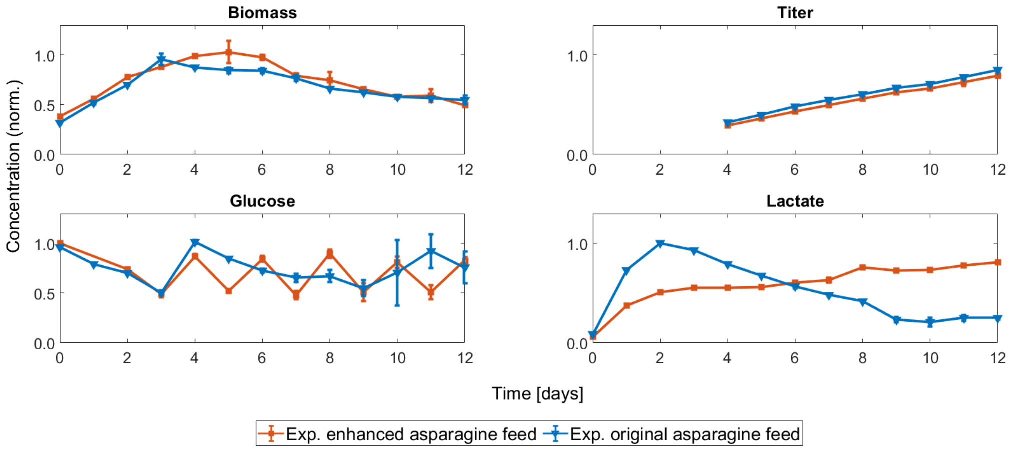

Figure S4. Measurements for experiments with the original and enhanced asparagine feed. Biomass, titer, glucose and lactate concentrations were measured for cultivations with the original asparagine content in the feed and an enhanced asparagine content in the feed for 12 days.
