# Supplementary material for: Deciphering Metabolic Pathways in High-Seeding-Density Fed-Batch Processes for Monoclonal Antibody Production: A Computational Modeling Perspective
Source: Bioengineering (Basel). 2024 Mar 28;11(4):331. doi: 10.3390/bioengineering11040331 (PMC11048072; doi:10.3390/bioengineering11040331)
Supplement: Supplementary file 1 [file bioengineering-11-00331-s001.zip › Supporting Material S1.pdf]

**Table S1.** List of reactions in the stoichiometric metabolic network.

| No. | Reaction |
|---|---|
| | **Glycolysis** |
| 1 | atp[c] + glc[c] -> adp[c] + g6p[c] + h[c] |
| 2 | g6p[c] <=> f6p[c] |
| 3 | atp[c] + f6p[c] -> adp[c] + f16p[c] + h[c] |
| 4 | f16p[c] <=> 2 g3p[c] |
| 5 | g3p[c] + adp[c] + p[c] + nad[c] <=> 3gp[c] + atp[c] + nadh[c] + h[c] |
| 6 | 3gp[c] <=> h2o[c] + pep[c] |
| 7 | adp[c] + h[c] + pep[c] -> atp[c] + pyr[c] |
| 8 | 2 h[c] + nadh[c] + pyr[c] <=> nad[c] + lac[c] |
| 9 | gtp[c] + oaa[c] -> co2[c] + gdp[c] + pep[c] |
| | **Pentose phosphate pathway** |
| 10 | 2 nadp[c] + h2o[c] + g6p[c] <=> 2 nadph[c] + 6pg[c] + h[c] |
| 11 | nadp[c] + 6pg[c] <=> nadph[c] + co2[c] + ri5p[c] |
| 12 | ri5p[c] <=> r5p[c] |
| 13 | ri5p[c] <=> x5p[c] |
| 14 | g3p[c] + s7p[c] <=> r5p[c] + x5p[c] |
| 15 | x5p[c] + e4p[c] <=> g3p[c] + f6p[c] |
| | **Tricarboxylic Acid Cycle (TCA)** |
| 16 | succoa[c] + acetoac[c] <=> suc[c] + acetoaccoa[c] |
| 17 | gtp[m] + h2o[m] --> gdp[m] + p[m] + h[m] |
| 18 | atp[m] + h2o[m] <=> adp[m] + p[m] + h[m] |
| 19 | fadh2[m] + fad[c] <=> fad[m] + fadh2[c] |
| 20 | pyr[m] + coa[m] + nad[m] --> accoa[m] + co2[m] + nadh[m] |
| 21 | mal[m] + nadp[m] -> pyr[m] + co2[m] + nadph[m] |
| 22 | pyr[m] + atp[m] + h2o[m] + co2[m] <=> oaa[m] + adp[m] + p[m] |
| 23 | accoa[m] + h2o[m] + oaa[m] <=> cit[m] + coa[m] + h[m] |
| 24 | cit[m] <=> isocit[m] |
| 25 | isocit[m] + nad[m] --> akg[m] + co2[m] + nadh[m] |
| 26 | akg[m] + coa[m] --> succoa[m] + co2[m] + h[m] |
| 27 | gdp[m] + p[m] + succoa[m] <=> gtp[m] + suc[m] + coa[m] |
| 28 | suc[m] + fad[m] <=> fum[m] + fadh2[m] |
| 29 | fum[m] + h2o[m] <=> mal[m] |
| 30 | mal[m] + nad[m] <=> oaa[m] + nadh[m] + h[m] |
| 31 | nadph[m] <=> nadp[m] + h[m] |
| 32 | nadh[m] <=> nad[m] + h[m] |
| | **Amino acid metabolism** |
| 33 | nad[c] + 3gp[c] + glu[c] + h2o[c] <=> h[c] + nadh[c] + ser[c] + akg[c] + p[c] |
| 34 | ser[c] --> nh4[c] + pyr[c] |
| 35 | ser[c] <=> gly[c] |
| 36 | cys[c] + h2o[c] + nad[c] <=> pyr[c] + nh4[c] + nadh[c] + h[c] |
| 37 | pyr[c] + glu[c] <=> ala[c] + akg[c] |
| 38 | h[c] + oaa[c] <=> co2[c] + pyr[c] |
| 39 | mal[c] + nad[c] <=> oaa[c] + nadh[c] + h[c] |
| 40 | coa[c] + atp[c] + cit[c] -> accoa[c] + adp[c] + p[c] + oaa[c] |
| 41 | fum[c] + h2o[c] <=> mal[c] |
| 42 | nad[c] + mal[c] -> nadh[c] + co2[c] + pyr[c] |

| | |
|---|---|
| 43 | atp[c] + propcoa[c] + hco3[c] <=> adp[c] + succoa[c] + p[c] + h[c] |
| 44 | akg[c] + val[c] + 3 nad[c] + o2[c] + fad[c] + coa[c] +2 h[c] <=> glu[c] + propcoa[c] + 2 co2[c] + 3 nadh[c] + fadh2[c] |
| 45 | thr[c] --> akbut[c] + nh4[c] |
| 46 | ser[c] + met[c] + atp[c] + 2 o2[c] + h[c] <=> cys[c] + akbut[c] + nh4[c] + amp[c] + pp[c] + co2[c] + h2o[c] |
| 47 | akg[c] + ile[c] + 2 nad[c] + fad[c] + 2 coa[c] + h[c] -> glu[c] + propcoa[c] + co2[c] + 2 nadh[c] + fadh2[c] + accoa[c] |
| 48 | akbut[c] + coa[c] + nad[c] <=> propcoa[c] + co2[c] + nadh[c] |
| 49 | phe[c] + nadh[c] + o2[c] + h[c] -> tyr[c] + nad[c] + h2o[c] |
| 50 | akg[c] + tyr[c] + h2o[c] + 2 o2[c] <=> fum[c] + glu[c] + acetoac[c] + co2[c] + 2 h[c] |
| 51 | akg[c] + leu[c] + atp[c] + nad[c] + fad[c] + hco3[c] + coa[c] <=> glu[c] + acetoac[c] + accoa[c] + adp[c] + nadh[c] + fadh2[c] + p[c] + co2[c] |
| 52 | thr[c] + coa[c] + nad[c] <=> accoa[c] + nadh[c] + h[c] + gly [c] |
| 53 | lys[c] + 2 akg[c] + nad[c] + h2o[c] + fad[c] <=> aoxo[c] + 2 glu[c] + nadh[c] + fadh2[c] |
| 54 | trp[c] + 3 h2o[c] + 3 o2[c] <=> ala[c] + co2[c] + aoxo[c] + nh4[c] + for[c] |
| 55 | aoxo[c] + coa[c] + 2 nad[c] + h2o[c] <=> acetoaccoa[c] + 2 nadh[c] + 2 co2[c] + 2 h[c] |
| 56 | acetoaccoa[c] + coa[c] <=> 2 accoa[c] |
| 57 | asp[c] + akg[c] <=> oaa[c] + glu[c] |
| 58 | asn[c] + h2o[c] --> asp[c] + nh4[c] |
| 59 | nh4[c] + atp[c] + glu[c] -> h[c] + adp[c] + p[c] + gln[c] |
| 60 | gln[c] + h2o[c] --> glu[c] + nh4[c] |
| 61 | gluysa[c] + nad[c] + h2o[c] --> glu[c] + nadh[c] + 2 h[c] |
| 62 | orn[c] + akg[c] --> gluysa[c] + glu[c] |
| 63 | arg[c] + h2o[c] --> orn[c] + urea[c] |
| 64 | pro[c] + nad[c] + h2o[c] --> gluysa[c] + h[c] + nadh[c] |
| 65 | his[c] + 4 h2o[c] <=> glu[c] + 2 nh4[c] + for[c] |
| 66 | glu[c] + nad[c] <=> akg[c] + nh4[c] + nadh[c] + h[c] |
| 67 | atp[c] + asp[c] + gln[c] + h2o[c] <=> amp[c] + asn[c] + glu[c] + pp[c] + h[c] |
| 68 | ser[c] + hocys[c] <=> cys[c] + nh4[c] + akbut[c] |
| 69 | asp[m] + akg[m] <=> oaa[m] + glu[m] |
| 70 | glu[m] + nad[m] + h2o[m] <=> akg[m] + nh4[m] + nadh[m] + h[m] |

**Transport reactions**

| | | | |
|---|---|---|---|
| 71 | succoa[m] <=> succoa[c] | 115 | suc[c] <=> suc[ext] |
| 72 | co2[m] <=> co2[c] | 116 | mal[c] <=> mal[ext] |
| 73 | nh4[m] <=> nh4[c] | 117 | accoa[c] <=> accoa[ext] |
| 74 | asp[m] <=> asp[c] | 118 | lys[ext] <=> |
| 75 | akg[m] <=> akg[c] | 119 | trp[ext] <=> |
| 76 | cit[m] <=> cit[c] | 120 | tyr[ext] <=> |
| 77 | accoa[m] <=> accoa[c] | 121 | phe[ext] <=> |
| 78 | suc[m] <=> suc[c] | 122 | asp[ext] <=> |
| 79 | mal[m] <=> mal[c] | 123 | leu[ext] <=> |
| 80 | pyr[m] <=> pyr[c] | 124 | asn[ext] <=> |
| 81 | for[c] <=> for[ext] | 125 | gln[ext] <=> |

| | |
|---|---|
| 82 | pyr[c] <=> pyr[ext] |
| 83 | lys[c] <=> lys[ext] |
| 84 | trp[c] <=> trp[ext] |
| 85 | tyr[c] <=> tyr[ext] |
| 86 | phe[c] <=> phe[ext] |
| 87 | asp[c] <=> asp[ext] |
| 88 | leu[c] <=> leu[ext] |
| 89 | asn[c] <=> asn[ext] |
| 90 | gln[c] <=> gln[ext] |
| 91 | glu[c] <=> glu[ext] |
| 92 | his[c] <=> his[ext] |
| 93 | pro[c] <=> pro[ext] |
| 94 | arg[c] <=> arg[ext] |
| 95 | ile[c] <=> ile[ext] |
| 96 | val[c] <=> val[ext] |
| 97 | met[c] <=> met[ext] |
| 98 | cit[c] <=> cit[ext] |
| 99 | thr[c] <=> thr[ext] |
| 100 | cys[c] <=> cys[ext] |
| 101 | ser[c] <=> ser[ext] |
| 102 | ala[c] <=> ala[ext] |
| 103 | nh4[c] <=> nh4[ext] |
| 104 | co2[c] <=> co2[ext] |
| 105 | gly[c] <=> gly[ext] |
| 106 | glc[c] <=> glc[ext] |
| 107 | hocys[c] <=> hocys[ext] |
| 108 | s7p[c] <=> s7p[ext] |
| 109 | urea[c] <=> urea[ext] |
| 110 | e4p[c] <=> e4p[ext] |
| 111 | lac[c] <=> lac[ext] |
| 112 | o2[c] <=> o2[ext] |
| 126 | glu[ext] <=> |
| 127 | his[ext] <=> |
| 128 | pro[ext] <=> |
| 129 | arg[ext] <=> |
| 130 | ile[ext] <=> |
| 131 | val[ext] <=> |
| 132 | met[ext] <=> |
| 133 | thr[ext] <=> |
| 134 | cys[ext] <=> |
| 135 | ser[ext] <=> |
| 136 | ala[ext] <=> |
| 137 | nh4[ext] <=> |
| 138 | co2[ext] <=> |
| 139 | gly[ext] <=> |
| 140 | hocys[ext] --> |
| 141 | lac[ext] <=> |
| 142 | glc[ext] <=> |
| 143 | s7p[ext] <=> |
| 144 | urea[ext] <=> |
| 145 | e4p[ext] <=> |
| 146 | o2[ext] <=> |
| 147 | cit[ext] <=> |
| 148 | for[ext] --> |
| 149 | pyr[ext] <=> |
| 150 | ri5p[ext] --> |
| 151 | suc[ext] <=> |
| 152 | accoa[ext] <=> |
| 153 | mal[ext] <=> |
| 154 | biomass[c] -> biomass[ext] |
| 155 | biomass[ext] --> |
| 156 | mAb[ext] -> |

| | | | |
|---|---|---|---|
| 113 | hco3[c] + h[c] <=> h2o[c] + co2[ext] | 157 | fum[c] <=> fum[ext] |
| 114 | ri5p[c] <=> ri5p[ext] | 158 | fum[ext] <=> |
| | **Energy maintenance** | | |
| 159 | h2o[c] + atp[c] -> h[c] + adp[c] + p[c] | | |
| 160 | gtp[c] + h2o[c] <=> gdp[c] + p[c] + h[c] | | |
| | **Biomass formation** | | |
| 161 | 0.282 g6p[c] + 0.073 r5p[c] + 1.15 o2[c] + 4.02 nadph[c] + 2.08 accoa[c] + 0.28 pro[c] + 23 h2o[c] + 32.258 atp[c] + 0.19 met[c] + 0.66 gly[c] + 0.236 phe[c] + 0.27 glu[c] + 0.568 ala[c] + 0.26 asn[c] + 0.105 cys[c] + 0.558 gln[c] + 0.43 ser[c] + 0.32 thr[c] + 0.31 arg[c] + 0.398 asp[c] + 0.12 his[c] + 0.54 leu[c] + 0.278 ile[c] + 0.49 lys[c] + 0.41 val[c] + 0.0318 trp[c] + 0.16 tyr[c] -> biomass[c] + 28.063 h[c] + 31.961 adp[c] + 31.961 p[c] + 0.297 pp[c] + 4.02 nadp[c] + 1.97 coa[c] + 0.19 co2[c] + 0.297 amp[c] + 0.06059 hocys[c] | | |
| | **Protein synthesis** | | |
| 162 | 5.68 ala[c] + 7.99 arg[c] + 6.68 asn[c] + 10.79 asp[c] + 6.09 cys[c] + 3.92 glu[c] + 4.83 gln[c] + 7.32 gly[c] + 5.09 his[c] + 2.09 ile[c] + 7.04 leu[c] + 7.30 lys[c] + 3.99 met[c] + 4.37 phe[c] + 1.88 pro[c] + 2.49 ser[c] + 3.17 thr[c] + 1.03 trp[c] + 4.51 tyr[c] + 3.76 val[c] -> mAb[c] | | |

* [c] indicates metabolites in the cytosol, [ext] indicates extracellular metabolites
