# Supplementary material for: Deciphering Metabolic Pathways in High-Seeding-Density Fed-Batch Processes for Monoclonal Antibody Production: A Computational Modeling Perspective
Source: Bioengineering (Basel). 2024 Mar 28;11(4):331. doi: 10.3390/bioengineering11040331 (PMC11048072; doi:10.3390/bioengineering11040331)
Supplement: Supplementary file 1 [file bioengineering-11-00331-s001.zip › Supporting Material S2.pdf]

Table S2. List of abbreviations of metabolites in the models.

Abbreviation	Metabolite	Abbreviation	Metabolite
3gp	3-phosphoglyceric acid	6pg	6-phosphogluconate
accoa	acetyl-CoA	acetoac	acetoacetate
acetoaccoa	acetoacetyl-CoA	adp	adenosine diphosphate
akbut	alpha-ketobutyrate	akg	alpha-ketoglutarate
ala	alanine	amp	adenosine monophosphate
aoxo		arg	arginine
asn	asparagine	asp	aspartate
atp	adenosine triphosphate	cit	citrate
co2	carbon dioxide	coa	Co-enzyme A
cys	cysteine	e4p	erythrose-4-phosphate
f16p	fructose-1,6-bisphosphate	f6p	fructose-6-phosphate
fad/fadh2	Flavin adenine dinucleotide (oxidized/reduced)	for	formate
fum	fumarate	g3p	glycerinaldehyde-3-phosphate
g6p	glucose-6-phosphate	gdp	guanosine diphosphate
glc	glucose	gln	glutamine
gtp	guanosine triphosphate	glu	glutamate
gluysa	glutamate semialdehyde	gly	glycerine
h	proton	h2o	water
his	histidine	hocys	homocysteine
ile	isoleucine	isocit	isocitrate
lac	lactate	lys	lysine
mal	malate	met	methionine
nad/nadh	nicotinamide adenine dinucleotide (oxidized/reduced)	nadp/nadph	nicotinamide adenine dinucleotide phosphate (oxidized/reduced)
nh4	ammonia	o2	oxygen
oaa	oxaloacetate	orn	ornithine
p/pp	phosphate	pep	phosphoenolpyruvate
phe	phenylalanine	pro	proline
propcoa	propionyl-CoA	pyr	pyruvate
r5p	ribose-5-phosphate	ri5p	ribulose-5-phosphate
s7p	sedoheptulose-7-phosphate	ser	serine
suc	succinate	succoa	succinyl-CoA
thr	threonine	tyr	tyrosine
val	valine	x5p	xylulose-5-phosphate

* Tables may have a footer.
