# Supplementary material for: Deciphering Metabolic Pathways in High-Seeding-Density Fed-Batch Processes for Monoclonal Antibody Production: A Computational Modeling Perspective
Source: Bioengineering (Basel). 2024 Mar 28;11(4):331. doi: 10.3390/bioengineering11040331 (PMC11048072; doi:10.3390/bioengineering11040331)
Supplement: Supplementary file 1 [file bioengineering-11-00331-s001.zip › Supporting Material S3.pdf]

Table S3. List of reactions in the kinetic central carbon models.

No.	Model 1	Model 2/3
1	0.355 glc + 2.534 pyr + 2.834 glu + 0.260 asn + 1.600 ser + 0.398 asp + 0.540 leu + 0.278 ile + 1.384 nh4 → biomass	0.355 glc + 2.918 pyr + 3.184 glu + 0.260 asn + 1.515 ser + 0.398 asp + 0.540 leu + 0.278 ile + 1.223 nh4 → biomass
2	8.560 pyr + 62.060 glu + 3.970 asn + 3.460 asp + 2.500 ile + 7.660 leu + 23.260 ser + 22.890 nh4 → mAb	71.550 glu + 3.970 asn + 3.460 asp + 2.500 ile + 7.660 leu + 24.500 ser + 19.390 nh4 → mAb
3	glc → 2 pyr	glc → 2 pyr
4	pyr ↔ lac	pyr ↔ lac
5	asn ↔ asp + nh4	asn ↔ asp + nh4
6	asp ↔ glu	asp ↔ glu
7	2 glu + glc → 2 ser	glc → 2 ser
8	ile → glu	ile → glu
9	leu ↔ glu	leu ↔ glu
10	ser → nh4 + pyr	ser → nh4 + pyr
11	pyr ↔ glu	2 pyr → glu
