# Supplementary material for: Deciphering Metabolic Pathways in High-Seeding-Density Fed-Batch Processes for Monoclonal Antibody Production: A Computational Modeling Perspective
Source: Bioengineering (Basel). 2024 Mar 28;11(4):331. doi: 10.3390/bioengineering11040331 (PMC11048072; doi:10.3390/bioengineering11040331)
Supplement: Supplementary file 1 [file bioengineering-11-00331-s001.zip › Supporting Material S4.pdf]

Table S4. List of ordinary differential equations and rate expressions for the three distinct models

ODE/rate expression

Model 1

$$\frac{dbiomass}{dt} = v_1 \cdot biomass - k_d \cdot biomass - D \cdot biomass, \quad (1)$$

$$\frac{dmAb}{dt} = v_2 - D \cdot mAb, \quad (2)$$

$$\frac{dpyr}{dt} = -2.534 \cdot v_1 - 8.560 \cdot v_2 + 2 \cdot v_3 - v_4 + v_{10} - v_{11} + D \cdot (pyr_i - pyr), \quad (3)$$

$$\frac{dglc}{dt} = -0.355 \cdot v_1 - v_3 - v_7 - m \cdot biomass + D \cdot (glc_i - glc), \quad (4)$$

$$\frac{dlac}{dt} = v_4 + D \cdot (lac_i - lac), \quad (5)$$

$$\frac{dnh4}{dt} = -1.384 \cdot v_1 - 22.890 \cdot v_2 + v_5 + v_{10} + D \cdot (nh4_i - nh4), \quad (6)$$

$$\frac{dasn}{dt} = -0.260 \cdot v_1 - 3.970 \cdot v_2 - v_5 + D \cdot (asn_i - asn), \quad (7)$$

$$\frac{dasp}{dt} = -0.398 \cdot v_1 - 3.460 \cdot v_2 + v_5 - v_6 + D \cdot (asp_i - asp), \quad (8)$$

$$\frac{dglu}{dt} = -2.834 \cdot v_1 - 62.060 \cdot v_2 + v_6 - 2 \cdot v_7 + v_8 + v_9 + v_{11} + D \cdot (glu_i - glu), \quad (9)$$

$$\frac{dile}{dt} = -0.278 \cdot v_1 - 2.500 \cdot v_2 - v_8 + D \cdot (ile_i - ile), \quad (10)$$

$$\frac{dleu}{dt} = -0.540 \cdot v_1 - 7.660 \cdot v_2 - v_9 + D \cdot (leu_i - leu), \quad (11)$$

$$\frac{dser}{dt} = -1.600 \cdot v_1 - 23.260 \cdot v_2 + 2 \cdot v_7 - v_{10} + D \cdot (ser_i - ser) \quad (12)$$

$$\frac{dV}{dt} = F_{in} - F_{out} \quad (13)$$

$$v_1 = v_{max,1} \cdot \frac{pyr}{K_{mpyr1}+pyr} \cdot \frac{glu}{K_{mglu1}+glu} \cdot \frac{asn}{K_{masn1}+asn} \cdot \frac{glc}{K_{mglc1}+glc} \cdot \frac{K_{dlac1}}{K_{dlac1}+lac} \cdot \frac{K_{dnh41}}{K_{dnh41}+nh4}, \quad (14)$$

$$v_2 = a_2 \cdot biomass + b_2 \cdot v_1 \cdot biomass, \quad (15)$$

$$v_3 = v_{max,3} \cdot \frac{glc}{K_{mglc3}+glc} \cdot \frac{asn}{K_{masn3}+asn} + v_{3,base}, \quad (16)$$

$$v_4 = v_{max,4f} \cdot \frac{pyr}{K_{mpyr4}+pyr} \cdot \frac{1}{1+\left(\frac{lac}{K_{ilac4}}\right)^{\gamma 4}} - v_{max,4r} \cdot \frac{lac}{K_{mlac4}+lac} \cdot \frac{1}{v_3}, \quad (17)$$

$$v_5 = v_{max,5f} \cdot \frac{asn}{K_{masn5}+asn} - v_{max,5r} \cdot \frac{asp}{K_{masp5}+asp} \cdot \frac{nh4}{K_{mnh45}+nh4}, \quad (18)$$

$$v_6 = v_{max,6f} \cdot \frac{asp}{K_{masp6}+asp} - v_{max,6r} \cdot \frac{glu}{K_{mglu6}+glu}, \quad (19)$$

$$v_7 = v_{max,7} \cdot \frac{glu}{K_{mglu7}+glu} \cdot \frac{glc}{K_{mglc7}+glc}, \quad (20)$$

$$v_8 = v_{max,8} \cdot \frac{ile}{K_{mile8}+ile}, \tag{21}$$

$$v_9 = v_{max,9f} \cdot \frac{leu}{K_{mleu9}+leu} - v_{max,9r} \cdot \frac{glu}{K_{mglu9}+glu}, \tag{22}$$

$$v_{10} = v_{max,10} \cdot \frac{ser}{K_{mser10}+ser}, \tag{23}$$

$$v_{11} = v_{max,11f} \cdot \frac{pyr}{K_{mpyr11}+pyr} \cdot \frac{K_{iasn11}}{K_{iasn11}+asn} - v_{max,11r} \cdot \frac{glu}{K_{mglu11}+glu}, \tag{24}$$

Model 2

$$\frac{dbiomass}{dt} = v_1 \cdot biomass - k_d \cdot biomass - D \cdot biomass, \tag{25}$$

$$\frac{dmAb}{dt} = v_2 - D \cdot mAb, \tag{26}$$

$$\frac{dpyr}{dt} = -2.918 \cdot v_1 + 2 \cdot v_3 - v_4 + v_{10} - 2 \cdot v_{11} + D \cdot (pyr_i - pyr), \tag{27}$$

$$\frac{dglc}{dt} = -0.355 \cdot v_1 - v_3 - v_7 - m \cdot biomass + D \cdot (glc_i - glc), \tag{28}$$

$$\frac{dlac}{dt} = v_4 + D \cdot (lac_i - lac), \tag{29}$$

$$\frac{dnh4}{dt} = -1.223 \cdot v_1 - 19.390 \cdot v_2 + v_5 + v_{10} + D \cdot (nh4_i - nh4), \tag{30}$$

$$\frac{dasn}{dt} = -0.260 \cdot v_1 - 3.970 \cdot v_2 - v_5 + D \cdot (asn_i - asn), \tag{31}$$

$$\frac{dasp}{dt} = -0.398 \cdot v_1 - 3.460 \cdot v_2 + v_5 - v_6 + D \cdot (asp_i - asp), \tag{32}$$

$$\frac{dglu}{dt} = -3.184 \cdot v_1 - 71.550 \cdot v_2 + v_6 + v_8 + v_9 + v_{11} + D \cdot (glu_i - glu), \tag{33}$$

$$\frac{dile}{dt} = -0.278 \cdot v_1 - 2.500 \cdot v_2 - v_8 + D \cdot (ile_i - ile), \tag{34}$$

$$\frac{dleu}{dt} = -0.540 \cdot v_1 - 7.660 \cdot v_2 - v_9 + D \cdot (leu_i - leu), \tag{35}$$

$$\frac{dser}{dt} = -1.515 \cdot v_1 - 24.500 \cdot v_2 + 2 \cdot v_7 - v_{10} + D \cdot (ser_i - ser), \tag{36}$$

$$\frac{dV}{dt} = F_{in} - F_{out} \tag{37}$$

$$v_1 = v_{max,1} \cdot \frac{pyr}{K_{mpyr1}+pyr} \cdot \frac{glu}{K_{mglu1}+glu} \cdot \frac{asn}{K_{masn1}+asn} \cdot \frac{glc}{K_{mglc1}+glc} \cdot \frac{K_{dlac1}}{K_{dlac1}+lac} \cdot \frac{K_{dnh41}}{K_{dnh41}+nh4}, \tag{38}$$

$$v_2 = a_2 \cdot biomass + b_2 \cdot v_1 \cdot biomass, \tag{39}$$

$$v_3 = v_{max,3} \cdot \frac{glc}{K_{mglc3}+glc} \cdot \frac{asn}{K_{masn3}+asn} + v_{3,base}, \tag{40}$$

$$v_4 = v_{max,4f} \cdot \frac{pyr}{K_{mpyr4}+pyr} \cdot \frac{1}{1+\left(\frac{lac}{K_{ilac4}}\right)^{\gamma_4}} - v_{max,4r} \cdot \frac{lac}{K_{mlac4}+lac} \cdot \frac{1}{v_3}, \tag{41}$$

$$v_5 = v_{max,5f} \cdot \frac{asn}{K_{masn5}+asn} - v_{max,5r} \cdot \frac{asp}{K_{masp5}+asp} \cdot \frac{nh4}{K_{mnh45}+nh4}, \tag{42}$$

$$v_6 = v_{max,6f} \cdot \frac{asp}{K_{masp6}+asp} - v_{max,6r} \cdot \frac{glu}{K_{mglu6}+glu}, \tag{43}$$

$$v_7 = v_{max,7} \cdot \frac{glc}{K_{mglc7}+glc}, \tag{44}$$

$$v_8 = v_{max,8} \cdot \frac{ile}{K_{mile8}+ile}, \tag{45}$$

$$v_9 = v_{max,9f} \cdot \frac{leu}{K_{mleu9}+leu} - v_{max,9r} \cdot \frac{glu}{K_{mglu9}+glu}, \tag{46}$$

$$v_{10} = v_{max,10} \cdot \frac{ser}{K_{mser10}+ser}, \tag{47}$$

$$v_{11} = v_{max,11f} \cdot \frac{pyr}{K_{mpyr11}+pyr} \cdot \frac{K_{iasn11}}{K_{iasn11}+asn}, \tag{48}$$

Model 3

$$\frac{dbiomass}{dt} = v_1 \cdot biomass - k_d \cdot biomass - D \cdot biomass, \tag{49}$$

$$\frac{dmAb}{dt} = v_2 - D \cdot mAb, \tag{50}$$

$$\frac{dpyr}{dt} = -2.918 \cdot v_1 + 2 \cdot v_3 - v_4 + v_{10} - 2 \cdot v_{11} + D \cdot (pyr_i - pyr), \tag{51}$$

$$\frac{dglc}{dt} = -0.355 \cdot v_1 - v_3 - v_7 - m \cdot biomass + D \cdot (glc_i - glc), \tag{52}$$

$$\frac{dlac}{dt} = v_4 - \frac{1}{Y_{\frac{lac}{biomass}}} \cdot biomass + D \cdot (lac_i - lac), \tag{53}$$

$$\frac{dnh4}{dt} = -1.223 \cdot v_1 - 19.390 \cdot v_2 + v_5 + v_{10} + D \cdot (nh4_i - nh4), \tag{54}$$

$$\frac{dasn}{dt} = -0.260 \cdot v_1 - 3.970 \cdot v_2 - v_5 + D \cdot (asn_i - asn), \tag{55}$$

$$\frac{dasp}{dt} = -0.398 \cdot v_1 - 3.460 \cdot v_2 + v_5 - v_6 + D \cdot (asp_i - asp), \tag{56}$$

$$\frac{dglu}{dt} = -3.184 \cdot v_1 - 71.550 \cdot v_2 + v_6 + v_8 + v_9 + v_{11} + D \cdot (glu_i - glu), \tag{57}$$

$$\frac{dile}{dt} = -0.278 \cdot v_1 - 2.500 \cdot v_2 - v_8 + D \cdot (ile_i - ile), \tag{58}$$

$$\frac{dleu}{dt} = -0.540 \cdot v_1 - 7.660 \cdot v_2 - v_9 + D \cdot (leu_i - leu), \tag{59}$$

$$\frac{dser}{dt} = -1.515 \cdot v_1 - 24.500 \cdot v_2 + 2 \cdot v_7 - v_{10} + D \cdot (ser_i - ser), \tag{60}$$

$$\frac{dV}{dt} = F_{in} - F_{out} \tag{61}$$

$$v_1 = v_{max,1} \cdot \frac{pyr}{K_{mpyr1}+pyr} \cdot \frac{glu}{K_{mglu1}+glu} \cdot \frac{asn}{K_{masn1}+asn} \cdot \frac{glc}{K_{mglc1}+glc} \cdot \frac{K_{dlac1}}{K_{dlac1}+lac} \cdot \frac{K_{dnh41}}{K_{dnh41}+nh4}, \tag{62}$$

$$v_2 = a_2 \cdot biomass + b_2 \cdot v_1 \cdot biomass, \tag{63}$$

$$v_3 = v_{max,3} \cdot \frac{glc}{K_{mglc3}+glc} \cdot \frac{asn}{K_{masn3}+asn} + v_{3,base}, \tag{64}$$

$$v_4 = v_{max,4f} \cdot \frac{pyr}{K_{mpyr4}+pyr} \cdot \frac{1}{1+\left(\frac{lac}{K_{ilac4}}\right)^{\gamma_4}} - v_{max,4r} \cdot \frac{lac}{K_{mlac4}+lac} \cdot \frac{1}{v_3}, \tag{65}$$

$$v_5 = v_{max,5f} \cdot \frac{asn}{K_{masn5}+asn} - v_{max,5r} \cdot \frac{asp}{K_{masp5}+asp} \cdot \frac{nh4}{K_{mnh45}+nh4}, \tag{66}$$

$$v_6 = v_{max,6f} \cdot \frac{asp}{K_{masp6}+asp} - v_{max,6r} \cdot \frac{glu}{K_{mglu6}+glu}, \tag{67}$$

$$v_7 = v_{max,7} \cdot \frac{glc}{K_{mglc7}+glc}, \tag{68}$$

$$v_8 = v_{max,8} \cdot \frac{ile}{K_{mile8}+ile}, \tag{69}$$

$$v_9 = v_{max,9f} \cdot \frac{leu}{K_{mleu9}+leu} - v_{max,9r} \cdot \frac{glu}{K_{mglu9}+glu}, \tag{70}$$

$$v_{10} = v_{max,10} \cdot \frac{ser}{K_{mser10}+ser}, \tag{71}$$

$$v_{11} = v_{max,11f} \cdot \frac{pyr}{K_{mpyr11}+pyr} \cdot \frac{K_{iasn11}}{K_{iasn11}+asn}, \tag{72}$$
