# Supplementary figures and images for: Deciphering Metabolic Pathways in High-Seeding-Density Fed-Batch Processes for Monoclonal Antibody Production: A Computational Modeling Perspective
Source: Bioengineering (Basel). 2024 Mar 28;11(4):331. doi: 10.3390/bioengineering11040331 (PMC11048072; doi:10.3390/bioengineering11040331)

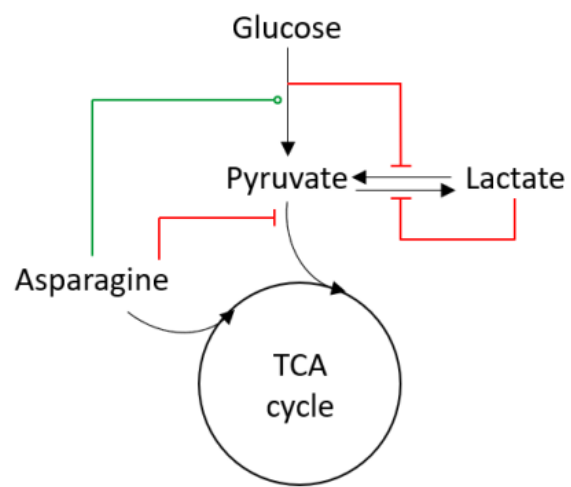

**Figure S2.** Visualization of the regulations behind the lactate shift in the dynamic model.

Supplement: Supplementary file 1 [file bioengineering-11-00331-s001.zip › Supporting Material Figure S2.pdf]
